# The Milk-Sickness of Upper Georgia and of Other Portions of the United States

**Published:** 1882-05

**Authors:** John H. Logan

**Affiliations:** Atlanta, Ga., Professor of Chcmistry in the Atlanta Medical College


### THE MILK-SICKNESS OF UPPER GEORGIA AND OF OTHER PORTIONS OF THE UNITED STATES.

By JOHN H. LOGAN, M.D., Atlanta, Ga.,
Professor of Chemistry in the Atlanta Medical College.

In the last issue of the Atlanta Medical Register there appeared an article on the mysterious disease whose name occupies the head of this paper, a thesis by Dr. K. H. Davis, of Dade county, Georgia, and a graduate from the Atlanta Medical College at its recent commencement.

Dr. Davis deserves no little credit for this timely contribution, brief as it is, on a subject so interesting in itself, and so largely related to the sanitary well-being of populous communities of our people. It is this importance of the subject, so little appreciated by the profession at large, few of whom having ever seen the malady in its native habitat, that serves me as a plea for indulgence in a more extended discussion of it.

But preliminary to this I must venture, with due modesty, to suggest a much needed improvement of the nomenclature of the disease. A name that signalizes no definite information as to the origin, nature or composition of a thing has no claims to be, in any sense, scientific. When we speak of cuprous sulphate, it is by a name and notation that is the perfection of scientific nomenclature. It indicates not only the substance itself, but its quantity, volume and composition as well.

The term milk-sickness falls too far short of such a standard. It conveys no adequate meaning or description of the thing signified, is inelegant in form and its corruption, milk-sick, so much in use among the people of the infected districts, is both a vulgarism and a violation of good grammar. It would be just as reasonable and lucid to talk of beef sickness, pig-sickness and butter-sickness, each one of these substances being as capable of imparting the disease as the infected milk.

It is quite time to correct this deficiency in the name of any disease that is worthy of a place in the books. A learned profession should be able to speak and write of the things with which it deals in at least the semblance of a scientific nomenclature.

In order to secure a scientific term, all that is necessary is to fix upon one or two essential characteristics of the thing to be named, and then to construct from these a simple or compound appellation. What, then, are the essential features of this disease called milk-sickness? One, and the chief, is that, according to nearly all observers, it is exclusively spontaneous in herbivorous animals. Another is, that these animals get it from no other source than the wild ranges on which they graze. These are quite enough for our purpose. What, now, shall be the combination? Let us go to the great storehouse of scientific nomenclature, the Greek. What is the Greek for the two essential words, pasture and evil, or sickness? It will be perceived that the word pasture implies not only the proximate source of the poison, but herbivorous animals as well, through whom it is transmitted.

The Greek word *nemos* means a wooded pasture, the very idea we want here, as will be seen in another place, while *kakos*, in the same language, conveys the idea of evil or bad. This word has already been long crystalized, as it were, in medical nosology in such words as cachexy, meaning bad habit, and cacotrophy, bad nutrition. The terms we need, therefore, are *kakos*, or *cacos*, as it is usually anglicized, and *nemos*; their combination being easy and resulting in a name at once scientific and euphoneous—*caconemia*—the pasture-evil or sickness. This is simple, and yet comprehensive enough to embrace all that is essential to the origin and associations of the disease.

*Caconemia*, then, is the name we modestly submit to the profession for this too long nondescript, or miscalled malady.

From Colonial times the geography of the pasture-evil or caconemia, has been mainly confined, in Tennessee, to the densely wooded and fertile valleys and mountain sides bordering the Elk and Duck rivers with their tributaries west of the Cumberland range and adjacent to Alabama and Georgia; in the latter State, to the western slopes, and westerly-exposed coves of the same mountains in the present territory of Dade and Walker counties. The most infected cove being that known as "Elmore's."

In western N. Carolina it is found in similar coves and on the wooded sides of the Smoky Mountains. A very intelligent medical student from that region of N. Carolina, informed me that in a single county there, there is a well-known range of nearly fifteen miles square infected by the organisms of this poison, where hundreds of cattle annually graze, every one of which is liable to be stricken by the disease.

It is a singular fact, that though East Tennessee and the portion of that State bordering on Kentucky, are exceedingly rough and mountainous, they are both perfectly free from the ravages of the caconemia poison. The districts where it still prevails, in Tennessee, N. Carolina and Georgia, as defined above, are remarkably productive, and in early times were covered with an exceedingly luxuriant growth of lofty trees and almost impenetrable underwood, affording one of the most prolific nurseries in the world for the poisonous exhalations or zymotic organisms so potent, as is now conceded, in the production of the most malignant types of epidemic disease.

It was these facts, coupled with the similarity of several symptoms of the caconemic malady to those of malignant bilious remittent fever, that caused Dr. W. H. Lea, who wrote in 1821, to fall into the erroneous belief that it is nothing more than a virulent form of that disease.

It is known now that caconemia is not confined to mountain ranges, river banks, and coves opening towards western winds and skies. It prevails with equal virulence and with the identical symptoms, on the undulating hills of southern Ohio and among the sunny prairies of Illinois and Indiana.

It would appear that the only essential factors to the production of the poison, are rich lands, associated with moisture, a certain, nicely adjusted point of temperature, and the alternations of darkness and light.

I also learn, from good authority, that it is not perhaps confined to the continent of America. Apjohn and Dunglison inform us that in France and Switzerland an acrid principle has been sometimes developed in milk, particularly that of the goat and sheep, which has produced inflammation and death. The specific contagion, however, must be different in these cases, since the symptoms given resemble more those of cholera than of our disease. The same writers also speak of a disease affecting certain cattle in Germany, which is known there under the name of milzbrand—spleen inflammation. It develops in the intestinal canal of persons who eat their flesh all the symptoms of an acrid poison.

*Caconemia* makes its appearance with a well marked periodicity; that is, every year between the months of June and October, manifesting most virulence in August and September. Dr. Gray, of Ohio, admits, however, that it may make its visits, at least occasionally, even in midwinter. The reason of this will be explained. Dr. Lea states that the primary poison may be so active as to attack both men and animals, after simple exposure to its vapor-like exhalations. Speaking of the origin of the disease, he says: "But be this as it may, there is less uncertainty on another point; men may be infected as other animals by similar exposure. Lying on the ground, in the poisonous tracts, or remaining there for several hours during the night, is always followed by an attack of this disease, which has occasionally been fatal." Dr. Gray denies this fact in the manifestations of the disease in Ohio. He writes: "Herbivorous animals are the first to be poisoned with it, and afterward it is transmitted to carnivorous animals, and man by their flesh and milk."*

All observers in the infected districts, singularly agree in their statements of many curious phenomena in the history of this disease. Dr. Davis in his thesis tells us that the people of Dade and Walker counties assert that they have known their cattle and horses poisoned by being fed with hay or fodder that had been gathered and housed from infected localities. Dr. Gray makes the same statement from Ohio. "It may"—speaking of the poison—"be gathered in hay and stored away, and fed to stock in winter, as has occurred in some well-authenticated instances."

It is also agreed that it is unsafe for stock to graze on infected grounds only early in the morning while the dew

*Transactions of the Ohio State Medical Society, 1877.

is still upon the grass, or during wet weather. A knowledge of this fact caused the farmers, at an early day, to pen their cattle at night, turning them out after nine or ten o'clock in the morning. As far back as 1821, they had run a fence many miles in length along the foot of the Cumberland in order to cut off this nuisance.

This last fact taken in connection with that of the dry, infected fodder from the barn, is certainly remarkable in their seeming contrariety. Why should the dry forage contain and impart the poison, while it is only the dry grass of the pasture or wild range that is free from it? We think they can be easily reconciled, and that they have an important connection with the true explanation of the nature of the poison.

They annihilate the whole group of venerable theories accounting for the disease; they are wholly at variance with the malarial fever hypothesis; they throttle, without benefit of clergy, the rhus toxicodendron and entire toxic-plant doctrine, and furnish not a vestige of foot-room for the decrepit telluric emanation theory.

But how are the facts reconciled? The microscopic, infinitesimal organisms, being developed under the conditions already named—fertile soil, moisture, light and darkness, prevailing winds and special temperature, are deposited upon the surrounding verdure of the stock ranges, together with the moisture of dew or rain. As the morning advances, or the sun grows hot, the moisture evaporates, and the organisms are borne with its vapor far away from the haunts of men or animals. The latter may now graze in safety, for the poison is gone.

Very different, doubtless, are the conditions under which the noxious fodder or hay is gathered. Tied in close bundles, or piled in large masses, in early morning, while still moist and teeming with myriads of the living germs of the specific poison, they are hauled and packed away in barns. Here in the darkness and mould they find a hospitable resting place, ready for future development in the stomachs of feeding animals.

The effects of heat, under the diurnal movements of the sun, upon the germs of this poison, as just noticed, bear an analogy to ordinary malaria too striking to be overlooked. Yet it is not these chiefly, but the symptomatic signs of the disease that have misled so many observers into the belief that it is only an intensified form of bilious remittent fever. Analogy, and not identity, is our objective point here.

It is a common belief in India that water is capable of absorbing malaria*. The same opinion is held by the orthodox in Georgia and South Carolina, and often utilized by placing sheets of water of considerable breadth between dwellings and sources of malarial poison. The evaporating dew-drops, conveying aloft in their aqueous vapor the germs of this disease, are alike the protection of cattle grazing upon the wild ranges of infected districts and of communities of people who live in close proximity to miasmatic emanations.

Malaria, it is well known, can be lifted in this way, in temperate zones, to a height of 500 feet. The primitive Indians of the South pitched their wigwams hard by rivers and creeks, to be convenient to fish and fertile lands, but they were too wise to place them upon bluffs and high grounds, in preference to the low, alluvial bottoms bordering the streams.

Another noticeable analogy is that the poison of this disease is effectually destroyed by cultivation. So is malaria. It would be waste of time to refer to the many instances in the South of the total disappearance of malarial diseases as one of the results of extended drainage and cultivation. Dr. M. S. Dixon, of Winchester, Tenn., says from personal observation in the districts of that State infected with this poison of caconemia, that cultivation of the poisonous tracts destroys their noxious quality. Dr. S. S. Gray, Treasurer of the Ohio Medical Society, remarks: "Cultivation destroys the poison." It must, therefore, be developed or propagated in the wild ranges on whose vegetation it is deposited.

There is another notable feature of resemblance pertinent to our purpose, between the two poisons. It is not

*Reynolds' System of Medicine, Vol. 1.

the case that all the stock-feeding in the same infected pasture are affected. Neither are all the members of a family who may have eaten of the same diseased beef or poisonous butter, stricken with the disease. A case in point in given in the report from the West.* A father and mother had become poisoned, but not so severely as to be unable to care for themselves, and not suspecting caconemia, they allowed a small child to subsist almost entirely for some days on butter and milk from the same cows from which they themselves had imbibed the disease; yet the child was not in the least affected. Why was this difference? It resulted simply from the peculiar nature of the poison. Why is galvanic electricity of low tension and constant current, while frictional electricity is of high tension and incontrolable discharge? Had the same family partaken together of the same dish poisoned by arsenic, or lead, or any vegetable alkaloid, all of them would have been affected and each with a severity proportioned to the amount of the noxious food ingested.

How often is it witnessed in the prevalence of epidemic diseases, as typhoid fever, small-pox and measles, that one or several members of a family are unaffected by the contagion, while the rest succumb to it. It can only be said of this peculiarity of the poisons of zymotic diseases, that such is their nature.

Franklin won scientific immortality by the mere identification of the electricity that thunders in the clouds, and that which crackles in the excited fur of a cat's back. And yet Franklin died without knowing anything more of the nature of electricity than the child born yesterday. It is a great stride towards the truth, and a practical knowledge of this or any disease, to be able to say confidently of its ultimate cause, it is not that, nor that, but this; and yet leave it to an indefinite future to define the nature of this cause.

Is the active poison of caconemia a zymosis? We will recur to this point in another place. There are phenomena enough, however, in the habitudes of this disease, which, while apparently quite dissimilar from all that is known

*Dr. S. S. Gray.

of malarial disorders, are, on a closer scrutiny, found to be in striking accord with them. Both Dr. Davis and the Secretary of the Ohio Medical Society agree in the statement that herbivorous animals are the first to be poisoned ; the disease being afterwards transmitted to man and carnivorous animals by eating their flesh, milk, butter or cheese. The Secretary affirms that not only are calves, pigs, dogs, cats and buzzards thus destroyed, but even fish have been known to die in a short time after eating of the dead animals.

This is very remarkable, and certainly has no exact analogies that we know of in malarial or epidemic diseases. But where, in the nature of things, is the material difference between a draught of water imparting specific disease through the stomach and alimentary canal in man, at least, and a certain quantity of infected butter cheese or roast beef. "And who can enumerate the instances in which almost entire families have been stricken with typhoid fever or diphtheria by drinking water from wells or cisterns in close proximity to leaky sewers and cesspools? The specific germ of one disease may find a congenial residence in certain liquids, while that of quite a different malady may find one in the oil of butter, cheese and the adipose tissue of muscle.

The symptoms of this singular affection are not the least interesting or remarkable part of its history. There is, first, languor, variable appetite, an indefinable gnawing at the stomach, nausea, torpidity of the bowels, stiffness of the limbs, and total disinclination to all muscular exertion. For this reason it is known in the West as the "slows." It is also called the "trembles," for if at this stage the patient be forced to exert himself vigorously, he instantly falls to trembling from head to foot, and now the nausea becomes agonizing, retching and vomiting. Dr. M. S. Dickson declares that this symptom resembles, more than anything else, the effects of a large dose of tartar emetic—the gnawing is augmented till the sensation at the stomach is that of intense burning, and the sluggishness of the bowels advances into a form of constipation that can be likened only to that of a mechanical obstruction.

Fever now begins to manifest itself, and the patient suffers from unquenchable thirst; his constant cry to physician and attendants is, "Water—more water;" yet only for a moment does this seem to cool the flame that is burning his stomach. The pain quickly returns, and the more water he drinks, the more are his sufferings increased. He tosses from one side of his bed to the other, seeking in vain for relief, and wildly throws the cover from his person, apparently utterly indifferent to, or unconscious of, exposure. The tongue is but slightly coated and moist; the breathing slow, the radial circulation almost normal, while, strange to say, that of the aorta is so violent as to elicit frequent complaints from the sufferer. There is no marked tenderness or pressure about the bowels. The matter first ejected from the stomach is white and glairy; it presently becomes muddy-brown. Sometimes, when the vomiting ceases, the patient has hiccough, and then he vomits again. The mind is clear to the end; and now his extremities become icy cold; a clammy sweat bathes his body, and the patient sinks into utter exhaustion and dies.

We have described a typical case; of course there are many in which some, perhaps most, of the above symptoms are wanting; but the nausea, vomiting, torpidity and constipation are ever, in some degree, present. And, in addition, a peculiar odor is perceptible about the patient, which is regarded as pathognomonic. Dr. Davis compares it to the smell of a calf's breath.

It is interesting to note the close agreement of the several observers of this disease, though so widely separated as to place and time. Dr. Lea thus briefly details the symptoms more than a half century ago, in Tennessee: "In men the disease is a gastritis; the stomach is extremely irritable, the bowels torpid and obstinately costive, with great febrile excitement and determination to the head. There is also a peculiar odor emanating from the patient, more especially as death approaches, which is, perhaps, the most striking diagnostic. I have seen cases attended, even, with black vomit, but the peculiar odor was wanting."

Dr. Davis agrees, too, in the main, both with the old details just given and those of the report from the West. He speaks, however, of a rapid, small, wiry pulse, high fever, excessive soreness of the bowels on pressure, and a pointed tongue with red edges. The last two observers, also, agree that convalescence from the disease is slow and relapses frequent, especially on exposure to heat, with considerable physical exertion. With some patients complete recovery is never attained; during the remainder of life, a little over-exertion brings back the nausea, trembling and exhaustion.

The prognosis is favorable, if the disease is met in time with the proper remedies. But there is no chemical nor empirical antidote known. The treatment of this mysterious scourge has been as various as the theories concerning its origin and nature, each practitioner adopting a course in accordance with his own peculiar views. First of all, it was impossible that such a malady could escape the keen scent of the vultures of quackery. They find in its mystery and rustic associations a never failing harvest of greed; and just as of old, invariably fortify themselves behind the proviso that their specific is a certain cure if taken in time. When the patient dies, it is equally certain that Dr. Belladony Gripsack was called in just a little too late. One of them, in Ohio, was discovered by a physician giving his patient whisky and lobelia.

The treatment laid down sixty years ago was as follows: Purge actively; to open the bowels is the *sine qua non;* but to effect this is extremely difficult. The most incredible doses of calomel and castor oil are sometimes given, aided by injections, without effect. This is to be followed up by active purgatives, blisters, diaphoretics, warm or cold applications, as the case may require, and sometimes the most powerful stimulants. In Georgia today, the treatment is scarcely more or less than the above. To open the bowels is the first and chief indication. While this is being effected by every possible device, a powerful adjuvant remedy is found in whisky and sweet oil, exhibited in doses of a tablespoonful of each every two hours.

The course pursued in Ohio is a little different in details, but the same in principle. Dr. Knouff relies chiefly on turpentine and whisky, and has seen much benefit from this remedy. Others rely on calomel followed by castor oil and turpentine. I would venture to suggest the addition of a sufficient quantity of opium to the calomel to relieve the strictured condition of the muscular coat of the bowels, and to aid at the same time in allaying the excessive nausea and pain at the stomach. The first and grand object is to arouse the secretions of the lower portion of the alimentary canal. This is best effected by citrate of magnesia with the free use of an enema of warm water, or of warm salt and water. These are to be often repeated till the bowels are emptied and relieved, and only desisted from when that object has been effected. A light, properly prepared diet will usually complete the cure.

Among the few intelligent observers of this disease, only one has reported an autopsy. This was held in Walker county, Georgia, by Dr. Green B. Gordon, in the case of a Miss ———, and is exceedingly unsatisfactory. The only valuable fact reported from it is that for three or four inches the small intestine had been united by adhesive inflammation. No examination was made, it seems, of the stomach or other organs. Physicians living in the infected districts should lose no opportunity to inspect by a post mortem the organic lesions of so serious and mysterious a disease.

We must now, in conclusion, recur to the subject of the true origin of *caconemia.* Is this disease one of the zymoses? It seems scarcely necessary to ask the question in view of the facts already presented. But if it be zymotic, what is the specific contagium vivum ? Here we approach the sharply defined line where the waves of scientific light are beating restive against the barriers of the outer darkness—where the advanced scientific thought of the age is manifesting an instinctive consciousness of the whispered utterances of a great revelation. No articulate sounds have yet been heard, but it is cheering to think that from this standpoint, so full of promise of future development, there is no step backward. There is consolation, too, in being privileged to add, that so soon as the National Board of

Health, or that of any State, shall have spoken demonstratively of the nature, structure and habitudes of the living organisms, specific in the production of yellow or malarial fever, I and others shall have much more that is interesting to write of the contagium vivum of *caconemia.*

